# The Effect of Novel Selenopolysaccharide Isolated from *Lentinula edodes* Mycelium on Human T Lymphocytes Activation, Proliferation, and Cytokines Synthesis

**DOI:** 10.3390/biom12121900

**Published:** 2022-12-19

**Authors:** Aleksander Roszczyk, Michał Zych, Katarzyna Zielniok, Natalia Krata, Jadwiga Turło, Marzenna Klimaszewska, Radosław Zagożdżon, Beata Kaleta

**Affiliations:** 1 Department of Clinical Immunology, Medical University of Warsaw, Nowogrodzka 59, 02-006 Warsaw, Poland; 2 Department of Immunology, Transplantology and Internal Diseases, Medical University of Warsaw, 02-006 Warsaw, Poland; 3 Department of Drug Technology and Pharmaceutical Biotechnology, Medical University of Warsaw, Banacha 1, 02-097 Warsaw, Poland

**Keywords:** immunomodulation, *Lentinula edodes*, T lymphocytes, PBMC

## Abstract

Polysaccharides isolated from *Lentinula edodes* are bioactive compounds with immunomodulatory properties. In our previous studies from *L. edodes* mycelium, we have isolated a selenium(Se)-enriched fraction (named Se-Le-30), a mixture of linear 1,4-α-glucan and linear 1,3-β- and 1,6-β-glucans. In this study, we analyzed the effects of Se-Le-30 on the activation and proliferation of human T lymphocytes stimulated by anti-CD3 and anti-CD3/CD28 antibodies (Abs) and on the production of cytokines by peripheral blood mononuclear cells (PBMCs). Se-Le-30 had effects on T cell proliferation induced by Abs against CD3 and CD28. It significantly inhibited the proliferation of CD3-stimulated CD4^+^ and CD8^+^ T cells and enhanced the proliferation of CD4^+^ T cells stimulated with anti-CD3/CD28 Ab. Moreover, Se-Le-30 downregulated the number of CD3-stimulated CD4^+^CD69^+^ cells, CD4^+^CD25^+^ cells, as well as CD8^+^CD25^+^ cells, and upregulated the expression of CD25 marker on CD4+ and CD8+ T cells activated with anti-CD3/CD28 Abs. Furthermore, Se-Le-30 enhanced the synthesis of IFN-γ by the unstimulated and anti-CD3/CD28-stimulated PBMCs, inhibited synthesis of IL-2 and IL-4 by CD3-stimulated cells, and augmented the synthesis of IL-6 and IL-10 by unstimulated, CD3-stimulated, and CD3/CD28-stimulated PBMCs. Together, we demonstrated that Se-Le-30 exerts immunomodulatory effects on human T lymphocytes. These observations are of importance for the prospective use of Se-Le-30 in research or as a therapeutic compound.

## 1. Introduction

*Lentinula edodes* (Berk.) Pegler (shiitake mushroom) is a source of numerous bioactive compounds of which the most valuable are polysaccharides [1]. *L. edodes*-derived polysaccharides, especially β-glucans, are biological response modifiers (BRMs) with anticancer, antimicrobial, and immunomodulatory properties [2]. Several randomized controlled studies demonstrated that fungal β-glucans exhibit immune-enhancing effects without causing noticeable adverse effects [3,4].

Lentinan is one of the widely studied polysaccharides from *L. edodes*. It is a highly purified β-(1→6) branched β-(1→3)-glucan with molecular weight (Mw) of 1.153 × 10^3^ g/mol, which is clinically used as an adjuvant in cancer therapies in some Asian countries [2]. 

It has been documented that the biological activity of fungal polysaccharides depends on their source (mycelium or fruiting bodies), methods of extraction, composition, Mw, branching degrees, and helical conformation [5,6,7]. Polysaccharides with higher Mw and triple helical conformation show stronger biological activity, which may be explained by their ability to induce clustering of various receptors [6,7,8]. Similarly, the sugar composition of polysaccharides affects their activity, for example, mannose, xylose, and arabinose have been related to immunomodulatory effects on macrophages [9]. 

So far, a number of surface receptors have been found to interact with β-glucans, including dectin-1 [10], complement receptor type 3 (CR3) [11,12,13,14], toll-like receptors (TLRs) type 2/4/6 [13,15,16,17,18], lactosyceramide (LaCer) [13,19], and scavenger receptors [13]. Thus, fungal polysaccharides, due to their ability of binding to multiple types of receptors on a wide variety of immune cells, depending on their composition, conformation, and Mw, may exhibit different biological effects and activate distinct signaling pathways.

In our previous studies, we isolated a selenium(Se)-enriched lentinan analog (named Se-Le-30) from *L. edodes* mycelial cultures. Structural studies demonstrated that obtained fraction is a mixture of linear 1,4-α-glucan and linear 1,3-β- and 1,6-β-glucans with much higher than lentinan Mw (3.62 × 10^6^ g/mol) [20]. We then analyzed the immunomodulatory properties of this glucan on healthy human peripheral blood mononuclear cells (PBMCs). We found that Se-Le-30 significantly inhibited the proliferation of PBMCs both when stimulated with anti-CD3 monoclonal antibody (mAb, OKT3) ad upon allostimulation in a mixed lymphocyte reaction (MLR), without causing cytotoxicity or reducing the TNF-α production by CD3^+^ T cells. These preliminary results suggested that Se-Le-30 is a T cell-selective immunosuppressant, which most likely acts through the modulation of signaling via the T cell receptor (TCR)/CD3 complex. While antibodies against CD3 effectively induce proliferation, however, in the absence of a costimulatory signal, proliferating T cells can lose their function or undergo early apoptosis [21]. Therefore, in the present study, we analyzed the effects of Se-Le-30 on the activation and proliferation of human T lymphocytes stimulated by CD3 and CD3/CD28 Abs and on the production of cytokines. 

## 2. Materials and Methods

### 2.1. Biosynthesis and Isolation of Se-Le-30

The *L. edodes* (Berk.) Pegler strain used in this study was American Type Culture Collection 48085 (ATCC, Manassas, VA, USA). Sodium selenite (Sigma, Saint Louis, MO, USA) was added to the culture medium to obtain a Se concentration of 30 μg/mL. *L. edodes* mycelium was cultivated under the conditions described in the previous papers [20,22,23,24]. The mycelium was harvested by filtration, washed, and freeze-dried, and Se-Le-30 was isolated by the modified Chihara method [25,26]. Its structural analysis was described in detail in our previous paper [20].

### 2.2. PBMCs Isolation

Blood samples were collected by venipuncture into heparin-coated tubes. PBMCs were isolated from 9 mL of whole blood using Histopaque-1077 (Sigma-Aldrich, Prague, Czech Republic) according to the manufacturer′s instructions. Blood samples were obtained from the Regional Blood Centre in Warsaw, Poland, under the approval of the Bioethics Committee of the Medical University of Warsaw (no. KB/174/2017; updated AKBE/186/2021).

### 2.3. Preparation for Flow Cytometry Analysis

Prior to flow cytometry assays, all antibodies, viability marker, and carboxyfluorescein succinimidyl ester (CFSE) were titrated to obtain the highest signal-to noise ratio for each fluorochrome. Fluorescence-Minus-One experiments were performed in order to determine the cut-off value for the positive population for each marker. Prior to conducting each experiment, an instrument quality control was performed. CFSE stock solution was prepared according to the manufacturer′s instructions and stored in aliquots of 5 µL at −20 °C for further use. 

### 2.4. Proliferation Assay

Isolated PBMCs were counted. Up to 1 × 10^7^ cells were washed, resuspended in 1 mL of RPMI 1640 medium (Gibco, Thermo Fisher Scientific, Waltham, MA, USA) with 5% heat-inactivated human serum (Sigma, Saint Louis, MO, USA) and transferred to a 14 mL Falcon tube. Next, 110 µL of Phosphate-Buffered Saline (PBS, Gibco, Thermo Fisher Scientific, Waltham, MA, USA) was added to 5 µL of CFSE stock solution. CFSE was added to the cells suspension, vortexed, and incubated at 25 °C, for 7 min in the dark. After incubation, cells were washed twice in PBS with 10% human serum. PBMCs (1 × 10^5^ cells/well) were cultured in 96-well flat-bottom plates (Greiner Bio-One, Kremsmünster, Austria) for 5 days in the following variants: (1) cells stimulated with Dynabeads coated with anti-CD3/CD28 Abs (ratio 2:5, Gibco, MA, USA); (2) cells stimulated with anti-CD3 Ab (plates were pre-coated with 0.75 μg/mL of Ab, BD Pharmingen, San Diego, CA, USA); and (3) unstimulated cells (control). PBMCs were incubated in the presence of Se-Le-30 (100 µg/mL) or without polysaccharide adding an equivalent amount of water for injection instead (control cultures). After 5 days, cells were collected and transferred to 4 mL polypropylene Falcon tubes, washed in PBS, and resuspended in 100 µL of 1:400 Zombie Violet™ stock solution (BioLegend, San Diego, CA, USA). Cells were incubated for 20 min at room temperature in the dark and washed with 2 mL BD Pharmingen Stain Buffer (BSA, BD Biosciences, San Jose, CA, USA). Next, they were resuspended in 100 µL of Stain Buffer, and labelled with anti-CD3, anti-CD4, and anti-CD8 Abs (15 min at room temperature in the dark). After incubation, cells were washed with 2 mL of Stain Buffer, resuspended in 100 µL of PBS with 0.01% sodium azide (Sigma, Saint Louis, MO, USA), and acquired with a DxFlex flow cytometer (Beckman Coulter, Brea, CA, USA) using CytExpert software (Beckman Coulter). Flow cytometry data were analyzed using FlowJo software (v. 10.8.1; BD, Ashland, OR, USA). The division index was calculated as the total number of divisions divided by the number of cells at the start of the culture. The proliferation index was calculated as the total number of divisions divided by the number of cells that went into division. The expansion index was calculated as the total number of cells divided by the number of cells at the start of the culture. The replication index was calculated as the total number of divided cells divided by the number of cells that went into division. 

### 2.5. Activation Assay

For the activation assay 2 × 10^5^ of PBMCs were seed on 96-well flat-bottom plates (Greiner Bio-One, Kremsmünster, Austria) and cultured for 12, 24, and 48 h in the following variants: (1) cells stimulated with anti-CD3/CD28 Abs (ratio 2:5, Gibco, MA, USA); (2) cells stimulated anti-CD3 Ab (coated on plate wells, 0.75 µg/mL, BD Pharmingen, USA); and (3) unstimulated cells (control cultures). PBMCs were incubated in the presence of Se-Le-30 (100 µg/mL) or without polysaccharide with an equivalent amount of water for injection. Cells were harvested after 12, 24, and 48 h, washed in 2 mL of Stain Buffer (BSA, BD Biosciences, San Jose, CA, USA), and labeled with mouse anti-human CD3-PerCP (Clone SK7, BD Biosciences, San Jose, California, USA), CD4-APC-Cy7 (Clone SK3, BD Biosciences, San Jose, CA, USA), CD8-APC (Clone SK1, BD Biosciences, San Jose, CA, USA), CD25-FITC (Clone 2A3, BD Biosciences, San Jose, California, USA), CD69-Brillant Violet 510 (Clone FN50, BioLegend, San Diego, CA, USA). PBMCs were incubated for 15 min at room temperature in the dark, washed in 2 mL of bovine serum albumin (BSA), and resuspended in 100 µL of PBS with 0.01% sodium azide. At least 2 × 10^4^ CD3^+^ T cells were acquired on Becton Dickinson FACSCanto II cytometer (BD FACSCanto II, Becton Dickinson, NJ, USA). Data were analyzed using BD FACS Diva 6.1.3. software. Cell surface expression of CD69 and CD25 (percentage) was analyzed in CD8^+^ and CD4^+^ T cells. For information on the gating strategy see Supporting Information (Figure S1). 

### 2.6. Multiplex Cytokine Profiling

Cytokines detection in cell culture supernatants was performed by Luminex^®^ Multiplex Assay. 2 × 10^5^ of PBMCs were cultured for 24 h in variants described above. Initially, dynabeads coated with CD3/CD28 Abs have been removed by a magnet. Cell cultures were transferred to 4 mL polypropylene tubes and centrifuged. Cell-free supernatants were recovered and stored at −80 °C for future analysis. 

Before analysis, Luminex calibration and verification were performed using MAGPIX Calibration Kit and MAGPIX Performance Verification Kit (Merck Millipore, Darmstadt, Germany). The concentration of IL-2, IL-4, IL-6, IL-10, and interferon (IFN)-γ was measured on MAGPIX (Merck Millipore, Darmstadt, Germany) with Luminex- based bead array MILLIPLEX^®^ Human Cyto Panel A (Merck Millipore, Darmstadt, Germany) according to manufacturer’s instructions. Standards and quality controls were run on the same plate as analyzed supernatants. Data were analyzed using xPONENT software (Luminex Corp., Austin, TX, USA).

### 2.7. Cell Viability Assay

On 96-well flat-bottom plates, 2 × 10^5^ of PBMCs were seeded (Greiner Bio-One, Kremsmünster, Austria) and cultured for 24 h at 37 °C in a humidified atmosphere with 5% CO_2_, in the presence of Se-Le-30 (100 µg/mL) and with an equivalent amount of medium and water for injection as controls. After incubation, cells were harvested, washed in 2 mL of PBS, resuspended in 100 µL of Zombie Violet™ (BioLegend, San Diego, CA, USA) stock solution at a ratio of 1:400, incubated for 20 min at room temperature in the dark, then washed with 2 mL of Stain Buffer and labeled with mouse anti-human CD3 Ab (CD3-PerCP, Clone SK7, BD Biosciences, San Jose, CA, USA) in 100 µL of Stain Buffer for 15 min. Next, cells were washed in 2 mL of Stain Buffer, resuspended in 100 µL of PBS wit 0.01% sodium azide, and acquired with DxFlex flow cytometer. For each variant, the percentage of CD3^+^ T lymphocytes positive for Zombie Violet dye was recorded and compared to control cultures.

### 2.8. Statistical Analysis

Statistical analysis of acquired data and their visualization were performed using GraphPad Prism 9.4.0 (GraphPad Software). The normality of the data sets distribution was tested using Kolmogorov-Smirnov, Shapiro-Wilk, Anderson-Darlin, D’Agostino, and Pearson tests. The data set was considered to have a normal distribution when each of the applied tests had a prediction value higher than 0.05. Determination for outliers was performed using both ROUT (Q = 1%) and Grubbs’ (α = 0.05) methods. The Student t-test was performed when the distribution of differences was normal, and the Wilcoxon test was used when the distribution of differences was not normal. A *p*-value of < 0.05 (*) was considered statistically significant, and *p* < 0.01 (**), or *p* < 0.001 (***) as highly significant. Graphs are presented as mean ± SEM (standard error of the mean). 

## 3. Results

### 3.1. Effects of Se-Le-30 on Human CD4^+^ and CD8 ^+^ T Cells Proliferation

**Figure 1:**
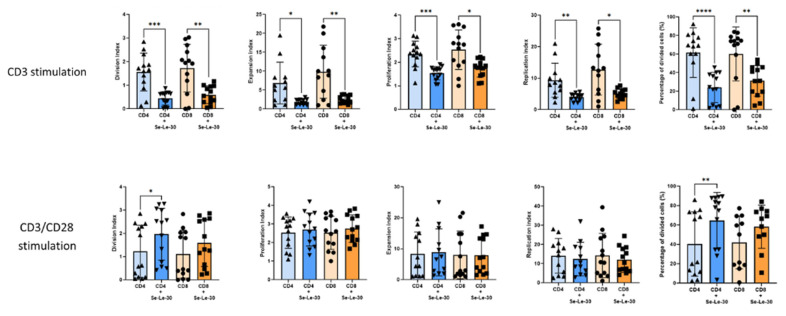
Effects of Se-Le-30 on the proliferation of human CD4^+^ and CD8^+^ T cells stimulated with anti-CD3 Ab (top row) and anti-CD3/CD28 Abs (bottom row). PBMCs from 13 healthy donors were stimulated and treated with Se-Le-30 (100 µg/mL) for 5 days. In each experiment, the division index, proliferation index, expansion index, replication index, and percentage of divided cells were calculated by using FlowJo software. Statistical differences were considered when *p* < 0.05. * *p* < 0.05; ** *p* < 0.01; *** *p* < 0.001; **** *p* < 0.0001. Points on bar charts represent experiments conducted with individual donors.

The effects of Se-Le-30 on the proliferation of human T cells are shown in Figure 1. Polysaccharide significantly inhibited the proliferation of CD4^+^ T cells stimulated with anti-CD3 Ab (division index: *p* = 0.0003; proliferation index: *p* = 0.0003; expansion index: *p* = 0.0141; replication index: *p* = 0.0095; percentage of cells divided: *p* < 0.0001) and enhanced proliferation when cells were stimulated with anti-CD3/CD28 Abs (division index: *p* = 0.0216; percentage of cells divided: *p* = 0.0033). Similarly, Se-Le-30 inhibited the proliferation of CD8^+^ T cells stimulated with anti-CD3 Ab (division index: *p* = 0.0012; proliferation index: *p* = 0.0123; expansion index: *p* = 0.0058; replication index: *p* = 0.0146; percentage of cells divided: *p* < 0.0001) but had no effect on double stimulated CD8^+^ T cells proliferation (*p* > 0.05). It must be noted that the mode of activation and possibly anti-CD3 Abs concentration was different for the stimulation with Abs against CD3 only and double stimulation with Abs against CD3 and CD28. 

### 3.2. Effects of Se-Le-30 on Human CD4^+^ and CD8 ^+^ T Cells Activation

Upon T cell activation, several of their cell surface markers are upregulated. CD69 is a very early marker, which can be detected on the surface of T cells 2–3 h after activation. CD25 (interleukin-2 receptor, IL-2R) is an early activation marker, with increased cell surface expression 12–24 h after activation [27]. 

**Figure 2:**
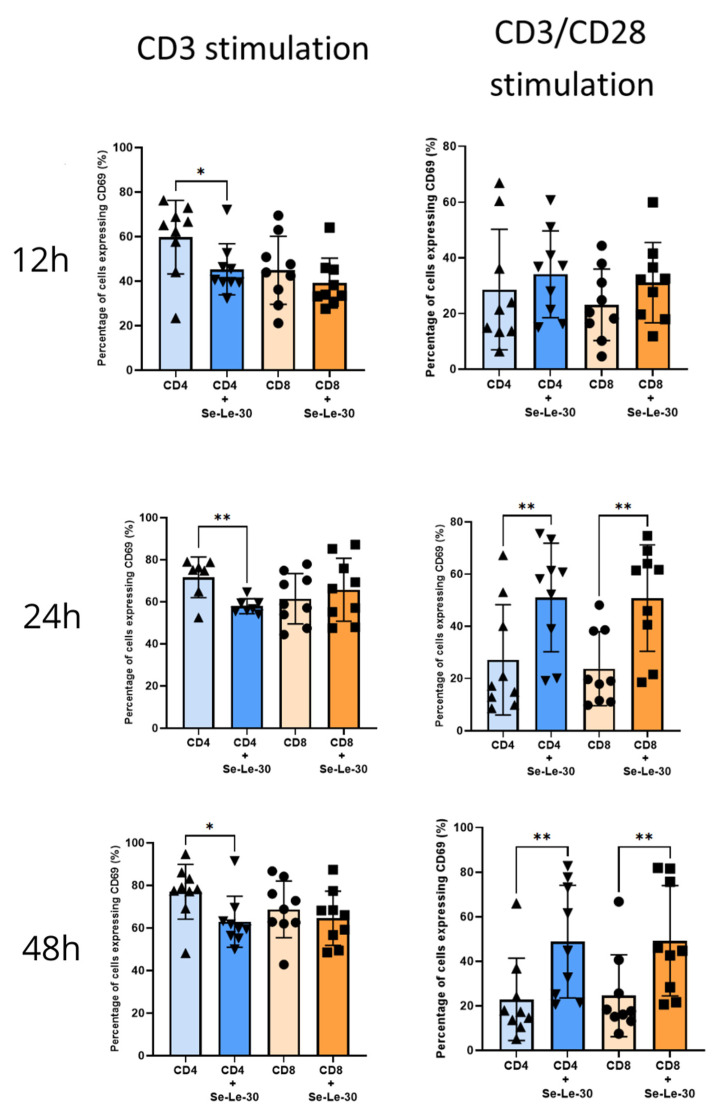
Effects of Se-Le-30 on human CD4^+^ and CD8^+^ T cells activation. PBMCs from nine healthy donors were stimulated with anti-CD3 Ab or anti-CD3/CD28 Abs and treated with Se-Le-30 (100 µg/mL) for 12, 24, or 48 h. The expression of the CD69 marker on the surface of CD4^+^ and CD8^+^ T cells was detected by flow cytometry. * *p* < 0.05; ** *p* < 0.01. Points on bar charts represent experiments conducted with individual donors.

The effect of Se-Le-30 on the presence of CD69 marker on T cells is presented in Figure 2. When cells were stimulated with anti-CD3 Ab, the polysaccharide significantly decreased the percentage of CD4^+^CD25^+^ T cells after 12, 24, and 48 h (*p* = 0.059, *p* = 0.0253, and *p* = 0.0219, respectively). When cells were stimulated with CD3/CD28 Abs, Se-Le-30 upregulated the percentage of CD4^+^CD69^+^ T cells after 24 and 48 h of culture (*p* = 0.0080, and *p* = 0.0015, respectively). The number of anti-CD3-stimulated CD8^+^ cells expressing the CD69 marker did not change after 12, 24, and 48 h of culture (*p* > 0.05), however, polysaccharide upregulated the percentage of anti-CD3/CD28-stimulated CD8^+^CD69^+^ T cells after 24 and 48 h of culture (*p* = 0.0065, and *p* = 0.0090, respectively).

**Figure 3:**
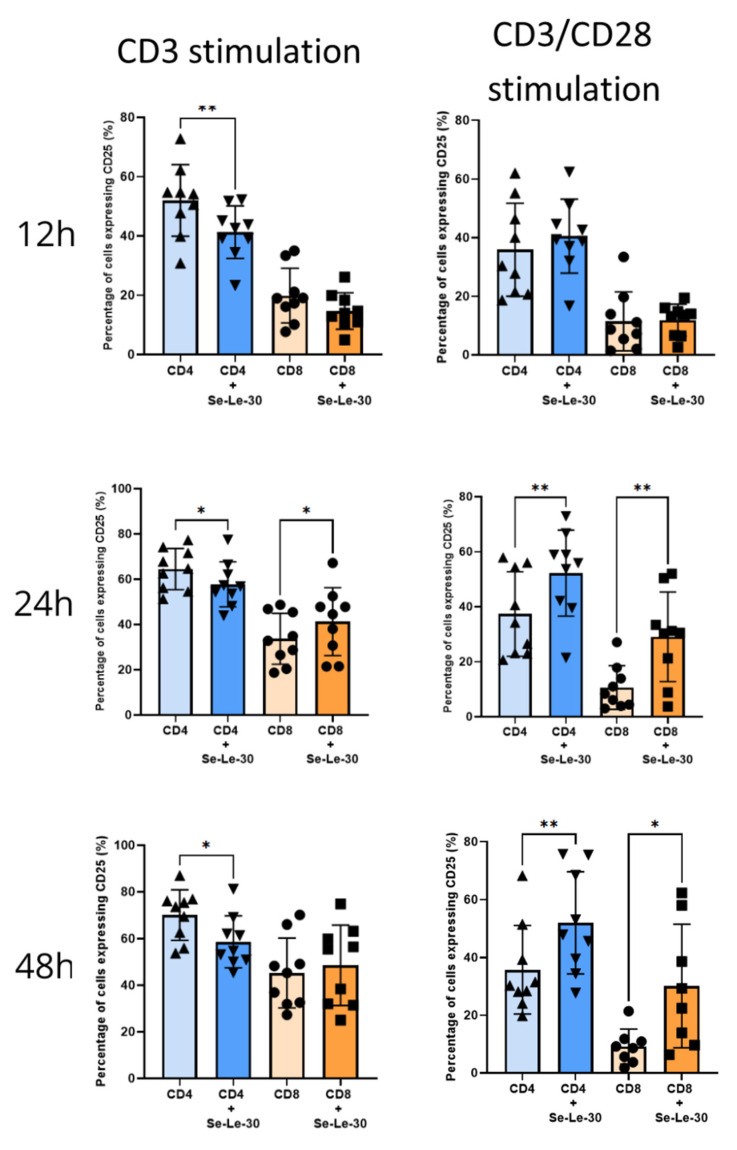
Effects of Se-Le-30 on human CD4^+^ and CD8^+^ T cells activation. PBMCs from nine healthy donors were stimulated with anti-CD3 Ab or anti-CD3/CD28 Abs and treated with Se-Le-30 (100 µg/mL) for 12, 24, or 48 h. The expression of the CD25 marker on the surface of CD4^+^ and CD8^+^ T cells was detected by flow cytometry. * *p* < 0.05; ** *p* < 0.01. Points on bar charts represent experiments conducted with individual donors.

The effect of Se-Le-30 on the presence of CD25 marker on T cells is presented in Figure 3. When cells were stimulated with anti-CD3 Ab, the polysaccharide significantly decreased the percentage of CD4^+^CD25^+^ T cells after 12, 24, and 48 h (*p* = 0.059, *p* = 0.0253, and *p* = 0.0219, respectively). When cells were stimulated with CD3/CD28, Abs Se-Le-30 increased the percentage of CD4^+^CD25^+^ T cells after 24 h and 48 h (*p* = 0.0032, and *p* = 0.0090, respectively). The number of anti-CD3-stimulated CD8^+^ cells expressing the CD25 marker did not change after 12 and 48 h of culture (*p* > 0.05) but slightly increased after 24 h of stimulation (*p* = 0.0501). Conversely, when cells were stimulated with anti-CD3/CD28 Abs, Se-Le-30 increased the percentage of CD8^+^CD25^+^ T cells (after 24 h: *p* = 0.0039, and after 48 h: *p* = 0.0095).

### 3.3. Effects of Se-Le-30 on Cytokines Production by PBMCs

**Figure 4:**
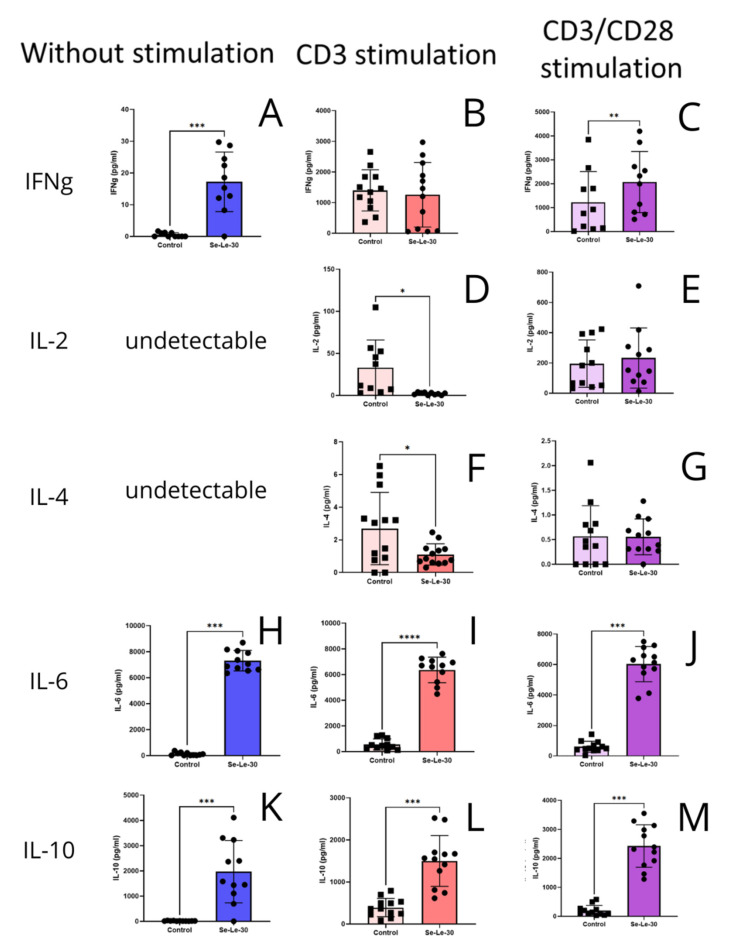
Effects of Se-Le-30 on cytokines secretion by PBMCs. PBMCs from 13 healthy donors were stimulated with anti-CD3 Ab and anti-CD3/CD28 Ab and treated with Se-Le-30 (100 µg/mL) for 24 h. The levels of secreted cytokines in the culture medium (pg/mL) were determined with a Luminex-based multiplex assay. (**A**–**C**): the levels of IFN-γ; (**D**,**E**): the levels of IL-2; (**F**,**G**): the levels of IL-4; (**H**–**J**): the levels of IL-6; (**K**–**M**): the levels of IL-10. Please note the differences in Y scale values between the stimulation panels, especially for IL-2. * *p* < 0.05; ** *p* < 0.01; *** *p* < 0.001; **** *p* < 0.0001. Points on bar charts represent experiments conducted with individual donors.

The effects of Se-Le-30 on cytokines secretion by PBMCs are shown in Figure 4. Polysaccharide had no influence on IFN-γ secretion by anti-CD3-stimulated PBMCs (*p* > 0.05), however, significantly upregulated its secretion in anti-CD3/CD28-stimulated cells (*p* = 0.0026). Moreover, it was observed that Se-Le-30 inhibited the secretion of IL-2 and IL-4 by anti-CD3-stimulated cells (*p* = 0.0170, and *p* = 0,0310, respectively), with no significant effect on anti-CD3/CD28-stimulated cells (*p* > 0.05). In addition, Se-Le-30 was found to increase the secretion of IL-6 and IL-10 by PBMCs in anti-CD3-stimulated and anti-CD3/CD28-stimulated cells (for IL-6 *p* < 0.0001, and *p* < 0.0001, respectively; for IL-10 *p* = 0.0003, *p* < 0.0001, respectively). 

### 3.4. Effect of Se-Le-30 on Human CD3^+^ T Cells Viability

**Figure 5:**
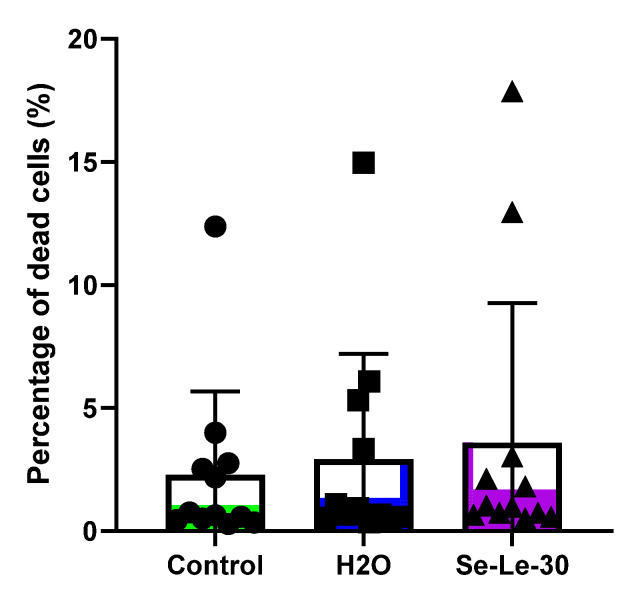
Effect of Se-Le-30 on CD3^+^ T cell viability. Evaluation of CD3+ T-cell viability after 24h culture with Se-Le-30 (100 µg/mL), with an equivalent amount of water for injection (H2O) or culture medium (control), was performed using Zombie Violet™ staining by flow cytometry. Cells were considered dead when they showed high violet fluorescence at 450 nm. Repeated measures one-way ANOVA test, *p* = 0.2076. Points on bar charts represent experiments conducted with individual donors.

The effects of Se-Le-30 on T cells viability are shown in Figure 5. Polysaccharide did not reduce CD3^+^ T cells viability after 24 h of culture. The percentage of dead cells did not differ statistically between control cultures and Se-Le-30 cultures. 

## 4. Discussion

Over the past few years, multiple studies have confirmed that various *L. edodes* polysaccharides can modulate the immune system through the activation of numerous signaling pathways. We have previously demonstrated that Se-Le-30, a selenium-enriched lentinan analog, significantly inhibited the proliferation of human T cells stimulated with anti-CD3 Ab [20,26,28]. In the present study, we have further compared the effects of Se-Le-30 on the activation and proliferation of human T lymphocytes stimulated by CD3 or CD3/CD28 Abs, as well as on their secretion of cytokines. The results of this study not only confirmed our previous observations regarding anti-CD3 Ab stimulation, but also demonstrated that when lymphocytes were stimulated with a dual signal (i.e., anti-CD3/CD28 Abs), Se-Le-30 enhanced their activation and proliferation: it increased the percentage of divided CD4^+^ T cells and their division index (please see Figure 1). These findings are in line with the results described by Wang et al. [29], who reported that lentinan, a β-1,3-branched β-1,6-D-glucan, increased the number of CD3^+^CD4^+^ and CD3^+^CD8^+^ T cells in patients with non-small cell lung cancer treated with chemotherapy. Moreover, this glucan inhibited the synthesis of IL-10, and enhanced IFN-γ. Conversely, our study indicated that Se-Le-30 increased the secretion of both IL-10 and IFN-γ in CD3/CD28-stimulated cells. In another randomized study, *L. edodes* was administered orally to healthy young adults and it was shown that fungus ingestion resulted in an increase in the proliferative potential of T lymphocytes, upregulated the expression of CD69 activation marker on T cells, and enhanced the production of IL-10 [30]. Similarly, it has been found that *L. edodes* extract consumption increased the plasma levels of IL-10 in healthy men exposed to exercise-induced skeletal muscle damage, but had no effect on the levels of IL-6 [31]. In the present study, we have found that Se-Le-30 increased IFN-γ secretion by PBMCs, which is consistent with data obtained in a study in patients receiving *L. edodes* mycelia extract combined with cancer immunotherapy [32]. Similarly, elevated serum IFN-γ levels were reported in a group of healthy adults administered rice bran fermented with *L. edodes* [32]. However, in contrast to our findings, there was no evidence of an effect of *L. edodes* on the regulation of IL-2, IL-4, and IL-10 secretion [33]. Another well-studied polysaccharide obtained from the mycelium of *L. edodes* is AHCC^®^, an active hexose-correlated compound which contains 20% of α-1,4-glucans [34]. Similar to Se-Le-30, AHCC^®^ was found to increase the secretion of IFN-γ and TNF-α by CD4^+^ and CD8^+^ T cells of healthy adults [35]. Moreover, this glucan increased the proliferation of CD8^+^ T cells in adults receiving the influenza vaccine [36]. 

On the other hand, some studies did not confirm the effect of *L. edodes* β-glucans on cytokines secretion in humans [37,38]. However, it should be noted that in these studies, the source of β-glucans was orally administrated substances, which might suggest that this type of administration might be ineffective to induce the effect of regulating the secretory activity of PBMCs.

Data on the immunomodulatory effects of glucans isolated from *L. edodes* in humans are limited, however, numerous studies have been conducted to evaluate its properties in animal models. Chen S. et al. demonstrated that three polysaccharide fractions with different molecular weights isolated from *L. edodes* reverted immune suppression in mice and upregulated splenic T lymphocytes proliferation in response to Concanavalin A and LPS [5]. The polysaccharide with the lowest molecular weight of 14-35 kDa was found to be the most effective [5]. In another study, the immunomodulatory properties of the synthetic analogue of lentinan basic unit- glucohexose have been analyzed. It was demonstrated that it upregulated CD69 expression on CD4^+^ and CD8^+^ T lymphocytes and increased the number of IFN-γ producing CD8^+^ T lymphocytes in mice [39]. These results are consistent with our study in humans. Most studies on the immunomodulatory properties of lentinan and its analogues have shown its immune-enhancing properties, however, there are some reports suggesting the immunosuppressive activity of *L. edodes* polysaccharides as well. McCormack and colleagues demonstrated that lentinan reduced serum levels of IL-4, IL-6, and IL-10 in rats [40]. Moreover, it was found that this drug enhanced the expansion of CD8^+^ T lymphocytes, which is similar to our findings in humans [41]. 

So far, numerous polysaccharide-binding receptors have been identified in immune cells, thus *L. edodes* polysaccharides can modulate the immune response by various signaling pathways. Dectin-1 is one of the most studied receptors responsible for the recognition of β-glucans and delivering activation signals [42]. Dectin-1 is expressed on numerous immune cells, including T and B lymphocytes, macrophages, dendritic cells, and neutrophils [10] and recognizes both β-(1→3) and β-(1→6) glucans [42]. Activation of dectin-1 by β-glucans stimulates phagocytosis, reactive oxygen species (ROS) production, NFκB-mediated cytokine secretion (including IL-1β, IL-2, IL-8, IL-10, IL-12, TNF-α), synthesis of chemokine CXCL2, differentiation of naive CD4^+^ T lymphocytes into Th1 and Th17, and activation of CD8^+^ T lymphocytes [43]. Complement receptor 3 (CR3) is expressed on neutrophils, monocytes, natural killer (NK) cells, CD8^+^ T cells, as well as activated CD4^+^ T cells and likewise has been implicated in the recognition of β-glucans [44,45]. Activation of CR3 upregulates phagocytosis and degranulation of cytotoxic CD8^+^ T cells [12]. TLRs are a family of pattern recognition receptors (PRRs) that recognize pathogen-associated molecular patterns (PAMPs) [46]. TLR2 is expressed on monocytes, neutrophils, B cells, and activated T cells [47] and acts as a costimulatory receptor that regulates cell proliferation [48,49]. TLR4 is highly expressed in monocytes [50], but is also involved in T cell development and differentiation [50,51]. TLR6 is mainly expressed on neutrophils and monocytes and is associated with NF-kappa-B activation and cytokines secretion [52,53]. LaCer is expressed on neutrophils and triggers cells response via NF-κB-like factor pathway, leading to oxidative burst [54] and production of macrophage inflammatory protein(MIP)-2 [55]. 

The binding of β-glucans to dectin-1 affects both CD8^+^ T lymphocytes and CD4^+^ T lymphocytes activation, resulting in enhanced granzyme production in CD8^+^ lymphocytes and differentiation of CD4^+^ into Th1 and Th17 phenotype [56,57]. Binding of β-glucans to CR3 results in the activation of phagocytes and NK cells, which promotes phagocytosis and cytotoxic degranulation. This may help to overcome tumor resistance to these forms of effector mechanism and lead to higher secretion of IL-6 and IFN-γ [12]. It has been suggested that 1,4-α-d-glucans do not affect macrophages via CR3 binding [58], but activation of T lymphocytes up-regulates expression of CR3 [44,45]. Interestingly, it was found that blocking the CD11b subunit of CR3 contributed to T lymphocyte proliferation inhibition after stimulation with anti-CD3 Abs [44]. Evidently, this finding allows us to hypothesize that Se-Le-30 might block CR3 and thus inhibit the proliferation of T lymphocytes when stimulated with anti-CD3 Ab only. Especially considering that CR3 expression on T lymphocytes is enhanced after activation. Dual CD3/CD28 stimulation would also be affected, but less significantly. This might be reflected in a higher division index and percentage of divided cells, while having no impact on other proliferation indexes.

There is growing evidence that polysaccharides derived from *L. edodes* interact with TLRs present in immune cells [15,16,59]. A study of 28 polysaccharides from different origins has shown that they interact with TLR4 and stimulate IL-10, while having no effect on IL-6 [18]. One recent study indicates that novel *L. edodes* polysaccharides (named MPSSS) may interact with cells via the TLR4/JNK pathway, which resulted in decreased secretion of vascular endothelial growth factor C (VEGF-C) [16] and/or interacts with TLR4-NF-κB pathway [15,60]. Another study on sparan, a 6-branched 1,3-β-D-glucan, demonstrated that this polysaccharide interacts with TLR4. Moreover, it was shown that sparan upregulated phosphorylation of ERK, p38, and JNK, and enhanced nuclear translocation of NF-κB p50/p65 in dendritic cells [59]. Indeed, (1,4)-α-d-glucans have been observed to interact with TLR6 receptors [58] and TLR4 in MyD88/IKK/NFκB pathway [61].

As mentioned above, dectin-1, CR3, and TLRs are implicated in the recognition of β-glucans by immune cells. Interestingly, recent research has shown that β-glucans may stimulate an immune response by binding to CD28 on the surface of T cells and that this stimulation is potentiated by CD3 activation. Cormer and colleagues explored interactions of β-1,3 glucans with the CD28 receptor and found that glucan molecules insert themselves into a channel on the surface of CD28 and moreover diffuse around the receptor, coming into contact with different regions of the protein [62]. This is another potential mechanism underlying the observed immunomodulatory effects of Se-Le-30. 

## 5. Conclusions and Future Perspectives

Se-Le-30, a fraction of polysaccharides isolated from mycelium of *L. edodes* is a mixture of linear 1,4-α-glucan and linear 1,3- and 1,6-β-glucans. In vitro models demonstrated that Se-Le-30 exerts immunomodulatory effects on human T lymphocytes, however, the direction of its biological activity depends on the type of cell stimulation. 

Moreover, it has been revealed that Se-Le-30 upregulated the production of IL-6 and IL-10 in PBMCs, regardless of the type of stimulation. Human PBMCs include lymphocytes, NK cells, monocytes, and dendritic cells, therefore, it is possible that other than T cell population is responsible for the observed activity. Future studies focusing on the mechanism of action of Se-Le-30, including identification of intracellular signaling pathways, as well as in vitro analyses with purified CD3^+^ T cells and other PBMC cell types are warranted.

## Data Availability

The data presented in this study are available on request from the corresponding author without any restrictions.

## Supplementary Materials

The following supporting information can be downloaded at: https://www.mdpi.com/article/10.3390/biom12121900/s1, Figure S1: Gating strategy.
