# Supplementary figures and images for: The Effect of Novel Selenopolysaccharide Isolated from Lentinula edodes Mycelium on Human T Lymphocytes Activation, Proliferation, and Cytokines Synthesis
Source: Biomolecules. 2022 Dec 19;12(12):1900. doi: 10.3390/biom12121900 (PMC9776057; doi:10.3390/biom12121900)

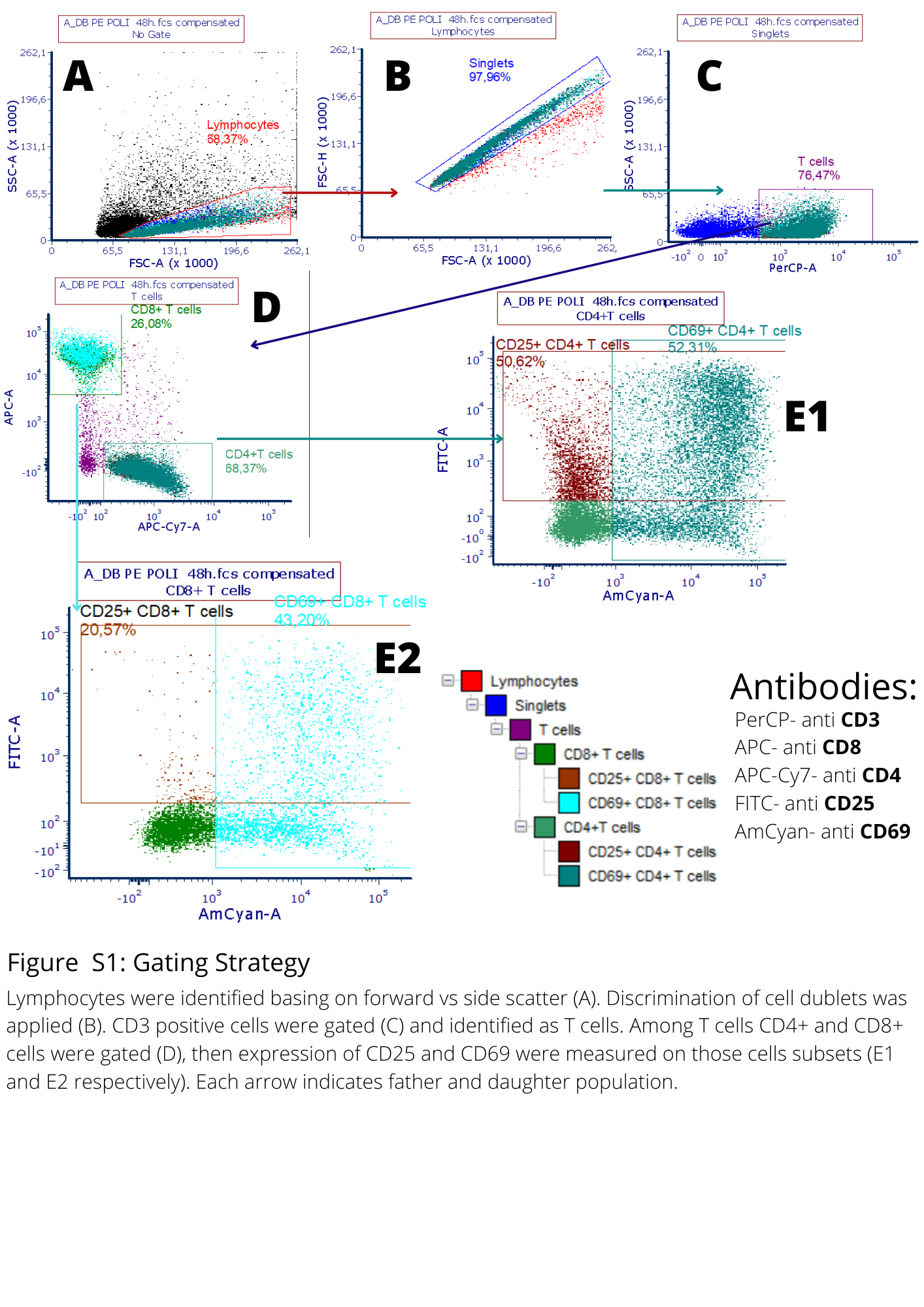

Supplement: Supplementary file 1 [file biomolecules-12-01900-s001.zip › biomolecules-2049334-supplementary.png]
